# Negative Pressure Wound Therapy Applied Before and After Split-Thickness Skin Graft Helps Healing of Fournier Gangrene: A Case Report (CARE-Compliant)

**DOI:** 10.1097/MD.0000000000000426

**Published:** 2015-02-06

**Authors:** Junna Ye, Ting Xie, Minjie Wu, Pengwen Ni, Shuliang Lu

**Affiliations:** From the Institute of Burns (JY, SL), Ruijin Hospital; and Department of Wound Healing (TX, MW, PN), Shanghai Ninth Hospital, School of Medicine, Shanghai Jiao Tong University, Shanghai, China.

## Abstract

Fournier gangrene is a rare but highly infectious disease characterized by fulminant necrotizing fasciitis involving the genital and perineal regions. Negative pressure wound therapy (NPWT; KCI USA Inc, San Antonio, TX) is a widely adopted technique in many clinical settings. Nevertheless, its application and effect in the treatment of Fournier gangrene are unclear.

A 47-year-old male patient was admitted with an anal abscess followed by a spread of the infection to the scrotum, which was caused by *Pseudomonas aeruginosa*. NPWT was applied on the surface of the scrotal area and continued for 10 days. A split-thickness skin graft from the scalp was then grafted to the wound, after which, NPWT utilizing gauze sealed with an occlusive dressing and connected to a wall suction was employed for 7 days to secure the skin graft.

At discharge, the percentage of the grafted skin alive on the scrotum was 98%. The wound beside the anus had decreased to 4 × 0.5 cm with a depth of 1 cm. Follow-up at the clinic 1 month later showed that both wounds had healed. The patient did not complain of any pain or bleeding, and was satisfied with the outcome.

NPWT before and after split-thickness skin grafts is safe, well tolerated, and efficacious in the treatment of Fournier gangrene.

## INTRODUCTION

Fournier gangrene (FG) is a rare but highly infectious disease characterized by fulminant necrotizing fasciitis involving the genital and perineal regions.^1^ This is one of the surgical emergencies with a high mortality rate of 40%.^2^ Early diagnosis of FG is vital to identify the need to start appropriate treatment. The principles of management are hemodynamic stability, broad-spectrum antibiotics, and prompt surgical debridement.

Negative pressure wound therapy (NPWT; KCI USA Inc, San Antonio, TX) is a popular treatment for the management of acute and chronic wounds. Its use is widespread amongst the surgical specialties, many of which employ NPWT to varying degrees as part of their armamentarium against challenging wounds.^3^ Negative pressure dressing was first described by Fleischmann et al in 1993.^4^ They noted that such a method had a positive effect on granulation tissue in open fractures. In 1997, Morykwas and Argenta^5^ studied the use of suction applied to polyurethane foam in wounds. The subatmospheric pressure was directed at the surface of the wound through an interface between the wound and a polyurethane sponge, allowing distribution of the negative pressure and use of either a constant or intermittent mode. NPWT is reported to be able to reduce wound volume where there has been tissue loss, promote granulation tissue formation, and reduce wound surface area with edge contraction.^6^ Moreover, it has been used to prepare wound beds for grafting or flap closure.^7^ Several studies have shown that NPWT can secure split-thickness skin grafts (STSG) and improve graft survival. However, in anatomically difficult body regions such as the perineum, it is questionable whether these dressings have similar beneficial effects.

In this case, the patient suffered from an anal abscess followed by a spread of the infection to the scrotum caused by *Pseudomonas aeruginosa*. We used NPWT before and after STSGs to help the wound healing.

## CASE REPORT

**FIGURE 1:**
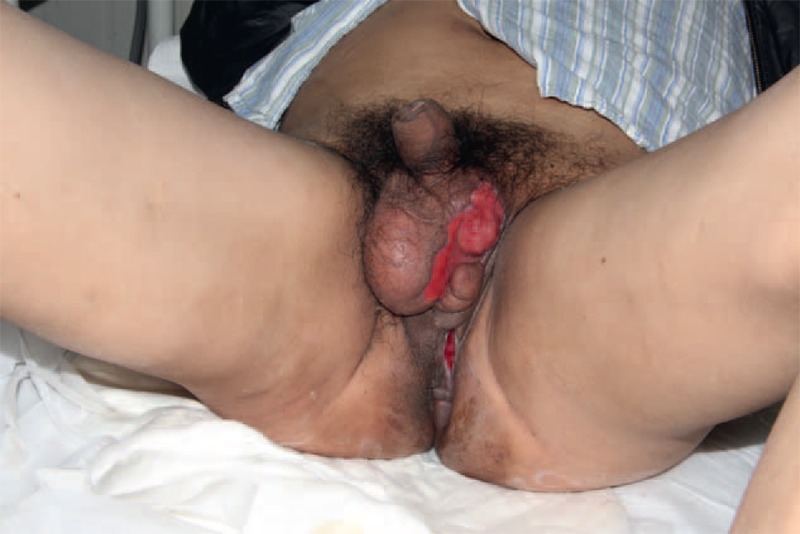
The patient suffered from FG initiated with an anal abscess. An incision and drainage was then emergently conducted. FG = Fournier gangrene.

**FIGURE 2:**
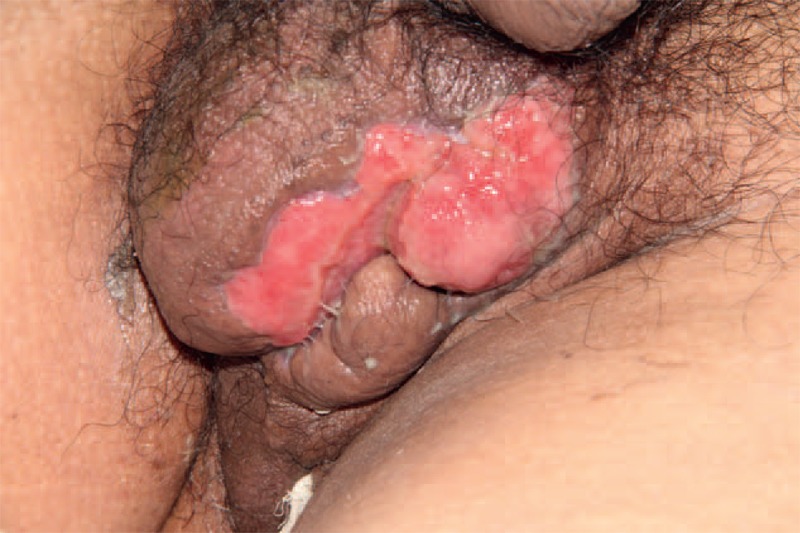
The infection caused by *P aeruginosa* spread from anal abscess to the scrotum. The defect of the scrotal skin was 6 × 7 cm, with pus on the surface.

A 47-year-old previously healthy male patient presented with fever (38.7°C), pain, erythema, and swelling around the anus. Examination showed the abscess area was 10 × 3 cm, with a depth of 10 cm. The swelling spread quickly to the region of left groin, perineum, and scrotum. There was associated erythema and edema of the scrotum and a poorly defined, perineal area of necrosis that discharged purulent liquid. No pain got on examination of the testes. Blood chemistry showed a white blood cell count of 17.92 × 10^9^/L (neutrophil percentage 86.7%), and a platelet count of 271 × 10^9^/L. He was immediately admitted to the local hospital and underwent emergent, extensive debridement in the operating room. However, despite the treatment, the skin of the scrotum kept necrotizing. So he was transferred to the Department of Wound Healing, a tertiary wound care center in Shanghai. The patient gave his informed consent prior to his inclusion in this study. On examination, the patient was alert and oriented but appeared unwell. Examination of the scrotum revealed a wound of 6 × 7 cm, which was discharging pus. The wound of the abscess beside the anus measured 4 × 1 cm with a depth of 5 cm. The pictures of his wounds are shown (Figures 1 and 2). Cultures of the exudates from the scrotal wound grew *P aeruginosa*. The patient remained afebrile with stable vital signs. There was no history of trauma and no symptoms of dysuria or hematuria. He had no history of diabetes, high blood pressure, or other chronic diseases. His past surgical history was unremarkable, and he was not on any regular medications. A review of systems was also unremarkable.

**FIGURE 3:**
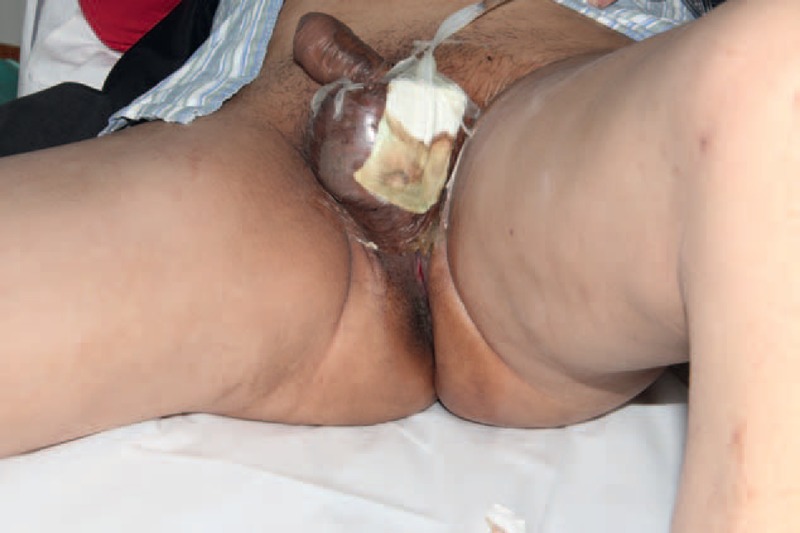
For the wound of scrotum, NPWT was applied and continued for 10 days before STSG. After STSG, the gauze was put on the scrotal wound and connected to the wall vacuum aspiration for 7 days. STSG = split-thickness skin graft.

The treatment after the hospitalization consisted of 2 stages. In stage 1, NPWT was applied to the surface of the scrotal area and continued for 10 days. After NPWT, the wound bed had a fresh red color. The exudates had lessened, and granulation tissue covered the surface of the wound. In stage 2, an STSG from his scalp was grafted to the scrotum. We gave the patient intravenous ofloxacin for 3 days after his STSG, to which he was sensitive. For economic consideration, we put gauze on the skin connected to the wall vacuum aspiration for 7 days. The gauze was changed every other day (Figure 3). During the usage of NPWT, the patient did not complain of any pain or bleeding, which were commonly seen complications. Then, the dressing was changed every 2 days using ionic silver foam dressings (Coloplast, Humlebæk, Denmark), whereas the wound beside the anus was treated with ionic silver alginate dressings (Coloplast).

**FIGURE 4:**
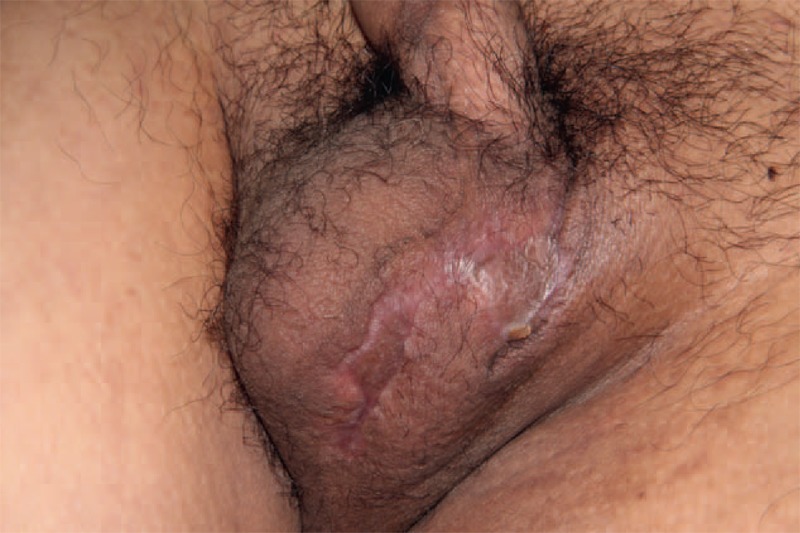
One month later when followed up, both the wounds of scrotum and anus had healed.

**FIGURE 5:**
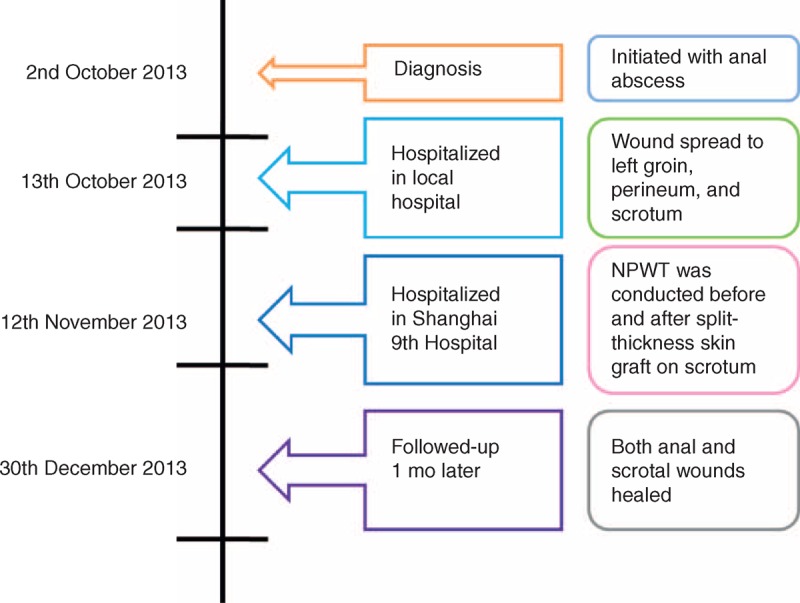
Timeline of interventions and outcomes.

After the treatment, the percentage of the grafted skin alive on the scrotum was 98%. The wound beside the anus had decreased to 4 × 0.5 cm with a depth of 1 cm. He was discharged from the hospital and followed up. The total time of hospitalization was 21 days. Follow-up at clinic 1 month later showed that both wounds had healed and that the patient was satisfied with the outcome (Figure 4). In addition, Figure 5 showed a timeline outlining the patient's interventions and outcomes.

## DISCUSSION

FG is a rapidly progressive necrotizing fasciitis of the perineum and external genital organs. Fournier described the first documented clinical picture of the disease in 1883.^8^ It can affect all age groups (mean age of 50) with a male predominance.^9^ The clinical appearance of FG is almost similar to cellulitis. The difference is that cellulitis is a surface infection without necrotic tissue, whereas microbes in Fournier disease cause infection of the small subdermal artery vessels leading to thrombosis.^10^ Arterial thrombosis causes ischemic necrotic fasciitis; however, muscle necrosis is rare. Here, we reported a case that was initiated by an anal abscess and spread to the perineal area with necrotizing scrotal skin. NPWT was then applied before and after STSG to help heal the wound.

There is evidence of NPWT being applied to various wounds. However, few are reported about its use in the scrotal area. The management of the perineal skin graft wound is complex for several reasons. Firstly, the perineum is close to the urethra anteriorly and the anus posteriorly, which can easily contaminate the wound. Secondly, the perineum has an irregular skin surface, preventing dressings applied to secure STSGs from exerting pressure evenly across the wound. Thirdly, the perineum is very mobile and therefore wound dressings can easily be dislodged.^11^

The skin from the scalp is easy to access and use in different parts of the body with a high possibility to be alive after transplantation. Therefore, we chose the scalp to be the donor site. In this case, NPWT was used prior to skin grafting in order to prepare the wound bed and afterwards to improve the chances of graft acceptance. Once the necrosis is eliminated, NPWT helps wound healing physiologically. The negative pressure leads to an increased blood supply and thus migration of inflammatory cells into the wound region.^12^ Moreover, NPWT after the skin graft can fix the wound and prevent the wound from further infection.

Although NPWT dressings and devices are more expensive than other wound-care products, cost-effective analysis shows lower treatment expenses. In a randomized controlled trial, Vuerstaek et al^13^ demonstrated significant shorter wound-preparation time and faster complete healing compared with the control group. They emphasized that higher treatment costs of the control group were created by higher personnel costs and longer hospitalization time due to slower healing. In this case, we applied the KCI materials before the STSG, and the negative pressure was set to be −125 mm Hg. As the patient could not afford KCI again, after his STSG, we used gauze connected to a wall vacuum system, which was much cheaper but functioned well. Additionally, Nguyen et al^14^ verified that a low-cost, readily accessible system utilizing gauze sealed with an occlusive dressing and wall suction (GSUC) results in comparable skin graft take compared with the vacuum assisted closure device. Therefore, for a cost-effective consideration, it may be beneficial to use a GSUC-based NPWT.

The bacteria that cause the destructive infection in FG are usually a mixture of causative microorganisms of colorectal origin. The debrided region is usually near the anus, which is prone to fecal contamination.^15^ Czymek et al^16^ compared data from 35 patients with FG. Twenty-eight of the 35 patients (80%) had polymicrobial infections. The most commonly isolated agents were *Escherichia coli* (n = 22, 62.9%), streptococcal species (n = 14, 40%), *P aeruginosa* (n = 9, 25.7%), and *Staphylococcus aureus* (n = 5, 14.3%). In this case, the infection of *P aeruginosa* was cultured, which was not so common. Ofloxacin was therefore prescribed intravenously for 3 days after his STSG. The vitals of the patient remained stable during hospitalization. At discharge, the patient had suffered minimal pain, and was satisfied with the outcome. NPWT helped heal the wound without causing other complications. Longer-term follow-up is necessary to further understand the functional and cosmetic results of the treatment.

## CONCLUSION

This case showed that NPWT before and after STSGs is safe, well tolerated, and efficacious in the healing of Fournier gangrene. However, more case series and randomized controlled trials are needed.
